# Stability and reproducibility comparisons between deep inspiration breath‐hold techniques for left‐sided breast cancer patients: A prospective study

**DOI:** 10.1002/acm2.13906

**Published:** 2023-01-23

**Authors:** David Parsons, Mindy Joo, Zohaib Iqbal, Andrew Godley, Nathan Kim, Ann Spangler, Kevin Albuquerque, Amit Sawant, Bo Zhao, Xuejun Gu, Asal Rahimi

**Affiliations:** ^1^ Department of Radiation Oncology University of Texas Southwestern Medical Center Dallas Texas USA; ^2^ Department of Radiation Oncology Inova Fairfax Hospital Falls Church Virginia USA; ^3^ Department of Radiation Oncology University of Maryland School of Medicine Baltimore Maryland USA; ^4^ Department of Medical Physics Memorial Sloan Kettering Cancer Center New York New York USA; ^5^ Department of Radiation Oncology Stanford University Palo Alto California USA

**Keywords:** breast, DIBH, SGRT

## Abstract

**Purpose:**

Deep inspiration breath‐hold (DIBH) is crucial in reducing the lung and cardiac dose for treatment of left‐sided breast cancer. We compared the stability and reproducibility of two DIBH techniques: Active Breathing Coordinator (ABC) and VisionRT (VRT).

**Materials and Methods:**

We examined intra‐ and inter‐fraction positional variation of the left lung. Eight left‐sided breast cancer patients were monitored with electronic portal imaging during breath‐hold (BH) at every fraction. For each patient, half of the fractions were treated using ABC and the other half with VRT, with an equal amount starting with either ABC or VRT. The lung in each portal image was delineated, and the variation of its area was evaluated. Intrafraction stability was evaluated as the mean coefficient of variation (CV) of the lung area for the supraclavicular (SCV) and left lateral (LLat) field over the course of treatment. Reproducibility was the CV for the first image of each fraction. Daily session time and total imaging monitor units (MU) used in patient positioning were recorded.

**Results:**

The mean intrafraction stability across all patients for the LLat field was 1.3 ± 0.7% and 1.5 ± 0.9% for VRT and ABC, respectively. Similarly, this was 1.5 ± 0.7% and 1.6 ± 0.8% for VRT and ABC, respectively, for the SCV field. The mean interfraction reproducibility for the LLat field was 11.0 ± 3.4% and 14.9 ± 6.0% for VRT and ABC, respectively. Similarly, this was 13.0 ± 2.5% and 14.8 ± 9% for VRT and ABC, respectively, for the SCV. No difference was observed in the number of verification images required for either technique.

**Conclusions:**

The stability and reproducibility were found to be comparable between ABC and VRT. ABC can have larger interfractional variation with less feedback to the treating therapist compared to VRT as shown in the increase in geometric misses at the matchline.

## 1 INTRODUCTION

For patients with left‐side breast cancer undergoing radiation therapy, the heart could receive a considerable amount of radiation dose due to the proximity to the target volume, thereby increasing the risk of radiation‐induced cardiac toxicity.^1^, ^2^ Deep inspiration breath‐hold (DIBH) during treatment delivery increases the separation between the heart and the chestwall and moves the heart inferiorly and away from the radiation fields, thereby, minimizing the influence of respiratory motion and reducing the mean heart dose by more than 50%.^3^ As a result, the risk of cardiac morbidity and mortality is significantly reduced as the rates of coronary events increase linearly with the mean heart dose by 7.4% per Gy.^2^ In addition, lung dose has been shown to be greatly reduced with DIBH.^4^, ^5^, ^6^

Several methods are available to monitor and/or control the respiratory motion. At our center, we have two clinical DIBH systems: Active Breathing Coordinator (ABC; Elekta AB, Crawley, UK) and VisionRT (VRT; VisionRT Ltd., London, UK). ABC is a spirometry‐based system designed to guide a patient's breath‐hold (BH). The active BH technique measures the respiratory volume and blocks the patient's airflow at a preset threshold value to induce a reproducible BH condition. The stability with ABC has been found to range between 1 and 2.1 mm in the three orthogonal directions.^7^ ABC has also demonstrated excellent intra‐ and inter‐fractional reproducibility of the chestwall and can significantly reduce the heart and lung doses.^8^, ^9^ The second DIBH system, VRT, is a surface‐guided technique. This method uses a passive technique in which the patient voluntarily takes and holds a DIBH. VRT then uses surface imaging to monitor and reduce localization uncertainty for setup and during irradiation by continuously comparing the patient's surface to a reference BH image. VRT has been shown to be a more accurate positioning method in comparison to the conventional laser and skin mark alignment.^10^, ^11^ The degree of setup reproducibility with VRT has been found to be approximately 2 mm.^12^ Combined with conformal cardiac blocking, VRT has been demonstrated to be an effective means to avoid cardiac perfusion defects.^13^

While DIBH provides substantial dosimetric benefits, it is also crucial that patients are positioned consistently and that BHs are stable and reproducible intra‐ and inter‐fractionally. As outlined above, studies have investigated the performance of ABC and VRT individually. However, these studies did not compare the two DIBH methods within the same patient population. In order to understand the accuracy of patient alignment at our institution, this study compares the intra‐ and inter‐fractional stability and reproducibility between the ABC and the VRT DIBH techniques for left‐sided breast patients. Each patient underwent whole‐breast radiotherapy treatment (WBRT) with both DIBH techniques.

This study focused on positioning and dosimetric variation along treatment beam delivery. The positioning study utilized megavoltage (MV) images acquired with an electronic portal imaging device (EPID) during treatment delivery to capture the patient's positional variations. The variations were evaluated based on the lung volume visible on the portal images. The dosimetry component focused on *in‐vivo* dosimetry measurements on patient's skin surface at several treatment fractions. The variation of the measured readings will provide further assessment of the reproducibility of each DIBH technique. In addition, we also recorded and compared ABC and VRT assisted DIBH treatment time.

## 2 MATERIALS AND METHODS

### 2.1 Patient data and treatment setup

An Institutional Review Board approved clinical study was designed to prospectively compare the intra‐ and inter‐fractional variations between ABC and VRT setups (NCT02694029; Active breathing coordinator‐based vs. VisionRT‐based DIBH for radiation for breast cancer). Eight patients with left‐sided breast cancer were included in this study, receiving WBRT with DIBH. Patients were treated to 5040 cGy in 28 fractions with the 3D conformal technique consisting of one anterior supraclavicular (SCV) field and two opposing tangential fields, medial and left lateral (LLat). The SCV field was half‐beam blocked using an asymmetric jaw, and the inferior tangential fields were matched to the inferior border of the SCV field. For each patient, half of the 28 fractions were treated with ABC while VRT was used for the other 14 fractions. The orders of the techniques were randomized. Note the original trial had 10 patients, but two were excluded since there was insufficient lung in the SCV and LLat field for analysis.

**FIGURE 1:**
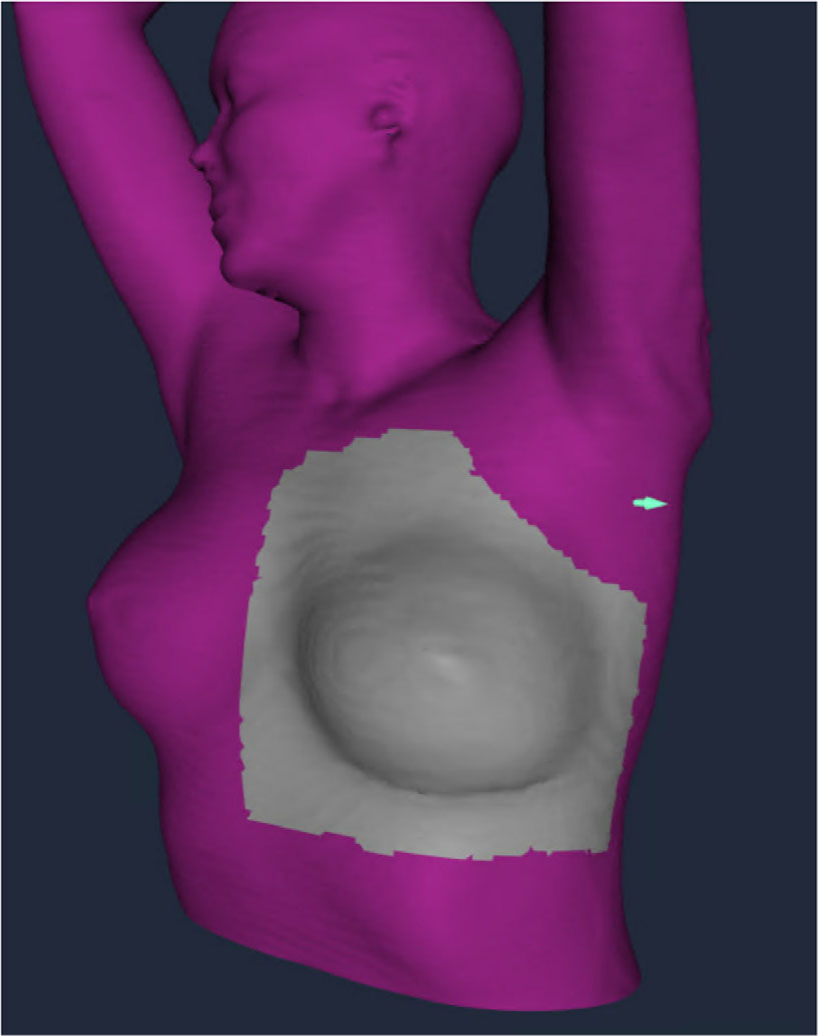
Example of VisionRT surface imaging with the region of interest around the left breast

The treatment room was equipped with a Varian Clinac 21EX (Varian Medical Systems, Inc, Palo Alto, California) with a gantry mounted EPID and VRT system. Setup verification and monitoring were performed according to our current clinical practice. For the ABC workflow, initial patient positioning was performed during free‐breathing (FB) by use of lasers and skin marks. For the fractions delivered with ABC, after the initial setup, patients were asked to take a DIBH, followed by pretreatment EPID images (at plan verification and once weekly). If setup deviations were observed, the treatment couch was adjusted accordingly and the patient re‐marked. For the VRT workflow, BH and FB body contours were extracted from the planning computed tomography (CT) scans obtained at simulation and imported into the VRT system as the reference images prior to the start of treatment. A region‐of‐interest (ROI) was outlined for each patient's scans in the software and defined as the surface region around the left breast (Figure 1). For the VRT fraction setup, patients were first positioned using lasers, skin marks, and the VRT FB surface. The magnitude of deviations, both translational and rotational, within the ROI between the setup and the reference surface was observed, and the patient and couch position were subsequently adjusted to minimize the deviation. Patients were then asked to perform a DIBH, and the BH contour from the BH CT images were used to evaluate the setup. Following the initial in‐room setup, EPID images were acquired while the patient was performing a DIBH for verification (at plan verification and once weekly).

Daily session time was recorded for both VRT and ABC treatments. Plan verify and weekly film days were recorded separately. Daily setup time was from patient entering the vault until finished the pretreatment imaging. Similarly, treatment time is from first treatment beam‐on until last beam‐off. Plan verify and weekly films session times were considered patient entering and leaving the vault. Additionally, the total imaging monitor units (MU) were recorded for patient positioning during these sessions.

### 2.2 Image acquisition and data analysis

**FIGURE 2:**
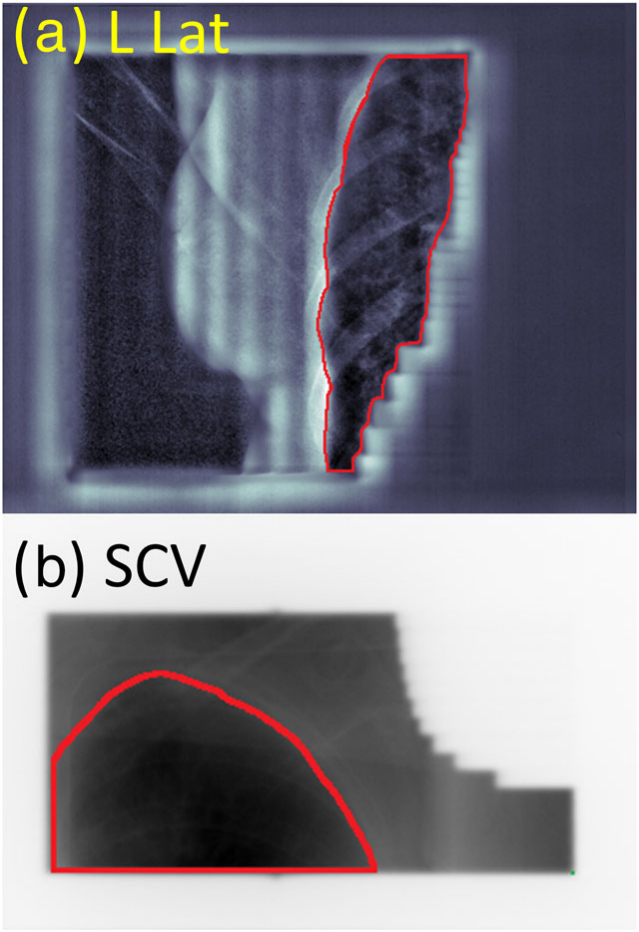
(a) LLat (with CLAHE filter) and (b) SCV MV image with a lung contour used to determine the lung area within the image

During every fraction, MV images in cine mode were acquired for each treatment field during irradiation. The images were exported and analyzed using MATLAB (The MathWorks, Inc., Natick, MA). The left lung was segmented in the images after application of a CLAHE filter and the lung area calculated at the isocenter plane. The SCV and LLat field images were used in this study. Additionally, images with modulation from the field‐in‐field technique were not included. Figure 2 shows an example of a LLat EPID image and the outline of the left lung. For each fraction, the coefficient of variation (CV) was calculated and determined to be the intrafraction variability (stability). Similarly, the CV for the lung area in the first image of each fraction represents the interfraction variability (reproducibility).

$$CV\;=\;100\frac{\sigma }{\bar{\mu }}$$

where σ is the standard deviation and $\bar{\mu }$ is the mean. Statistical analysis (Student's *t*‐test) was performed to determine the significant difference between the two DIBH techniques.

### 2.3 Dosimetry study

Five additional patients were included in a qualitative dosimetry study to examine the consistency of the SCV and tangent junction dose for DIBH. All patients were treated with a half‐beam blocked 3D technique and DIBH to the whole breast and the SCV region. Treatments were prescribed to either 5000 cGy in 25 fractions or 5040 cGy in 28 fractions. The treatment fractions were evenly divided into ABC and VRT as described above. Dose measurement was performed using radiochromic film (EBT3, Ashland Specialty Ingredients, Bridgewater, NJ) placed on the patient's skin and over the junction. The dose was normalized by OSLD dosimeters (nanaDotTM; Landauer, Glenwood, IL) placed on either side of the superior‐inferior junction. For each patient, measurements were done for both ABC and VRT fractions. Stability of the junction was then scored as either stable or potentially unstable. A potentially unstable junction was considering anything in which the gradient between the SCV and tangents had a gap greater or equal to 3 mm.

## 3 RESULTS

### 3.1 Lung intrafraction stability

**FIGURE 3:**
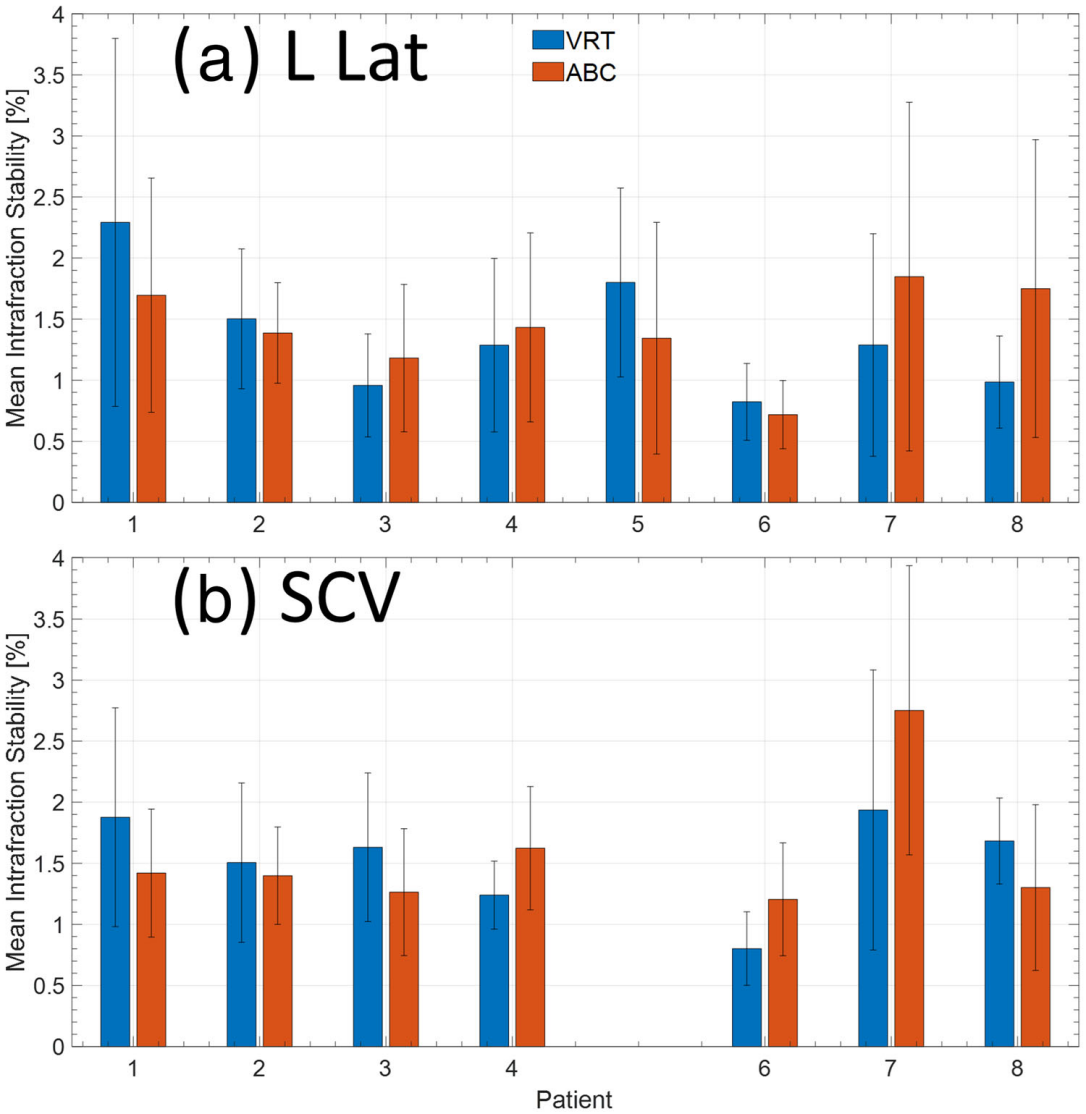
Mean intrafraction stability of the lung within the projection for the (a) left lateral and (b) SCV fields. The SCV was not included for patient 5 as the amount of lung within the MV projection was too small for accurate analysis

A total of 1828 EPID images sets for the eight patients were analyzed. Patient 5 was not included in the SCV analysis due to the lung area being too small for accurate analyze as very small changes (<10 mm^2^) in area resulted in a large change to the stability within a fraction. Figure 3 compares the mean lung area intrafraction stability in the LLat and SCV field for each patient for both ABC and VRT. Among the patients analyzed, no clear trend is present as both ABC and VRT preformed comparably and generally within the error of one another within a given fraction. The mean intrafraction stability across all patients for the LLat field was 1.26 ± 0.67% and 1.46 ± 0.92% for VRT and ABC, respectively (n.s., *p* = 0.76). Similarly, this was 1.52 ± 0.70% and 1.55 ± 0.78% for VRT and ABC, respectively (n.s., *p* = 0.83), for the SCV field. There was no statistically significant difference among these results.

### 3.2 Lung interfraction reproducibility

**FIGURE 4:**
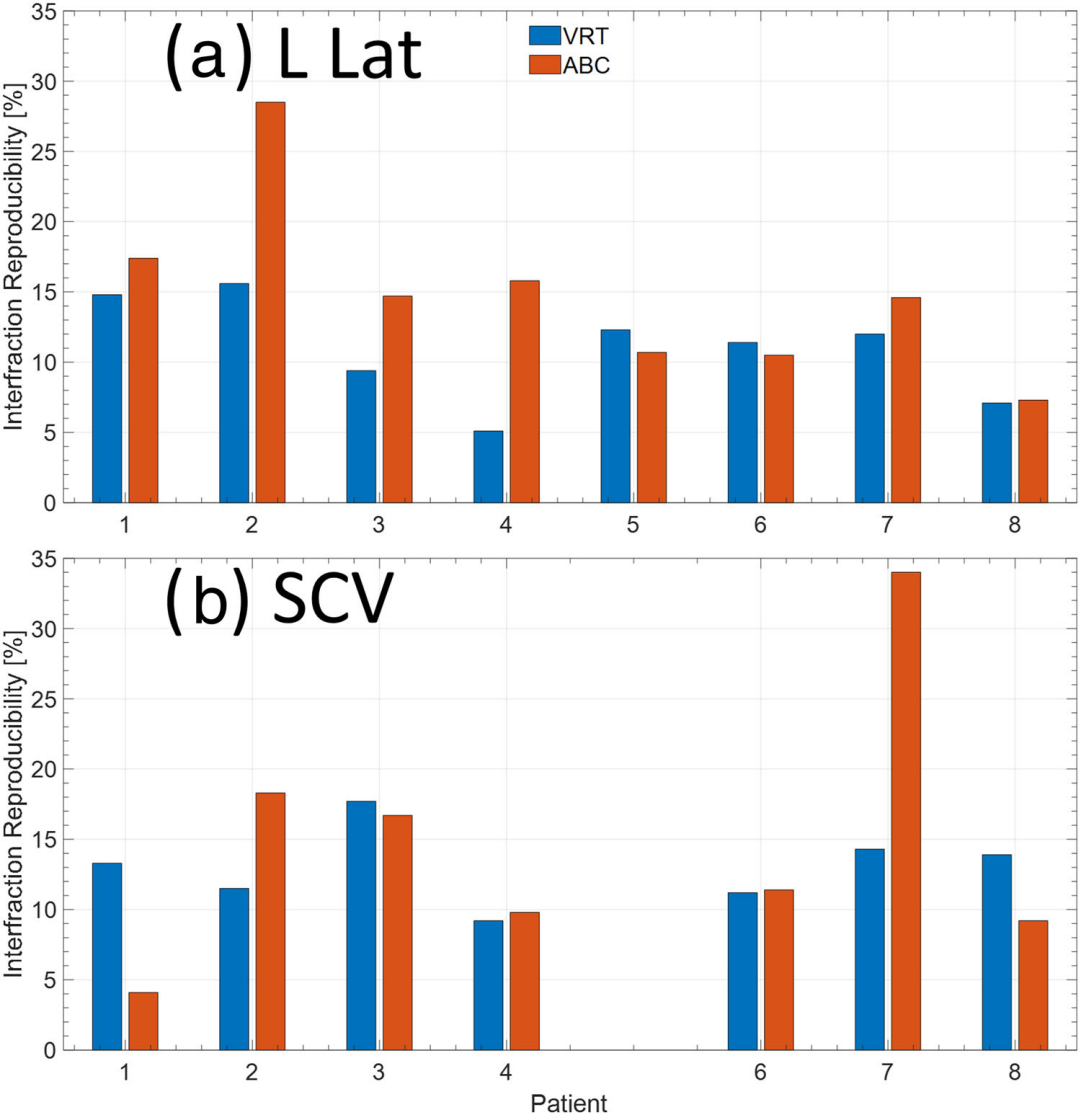
Interfraction reproducibility of the lung within the projection for the (a) left lateral and (b) SCV fields. The SCV was not included for patient 5 as the amount of lung within the MV projection was too small for accurate analysis

Figure 4 compares the interfraction lung area reproducibility in the LLat and SCV field for each patient for both ABC and VRT. The mean interfraction reproducibility across all patients for the LLat field was 11.0 ± 3.4% and 14.9 ± 6.0% for VRT and ABC, respectively (n.s., *p* = 0.07). Similarly, this was 13.0 ± 2.5% and 14.8 ± 9% for VRT and ABC, respectively (n.s., *p* = 0.63), for the SCV field. There was no statistically significant difference among the SCV result, while the LLat field is on the edge of being significant and would require additional patients to confirm.

### 3.3 Film study of junction dose stability

**FIGURE 5:**
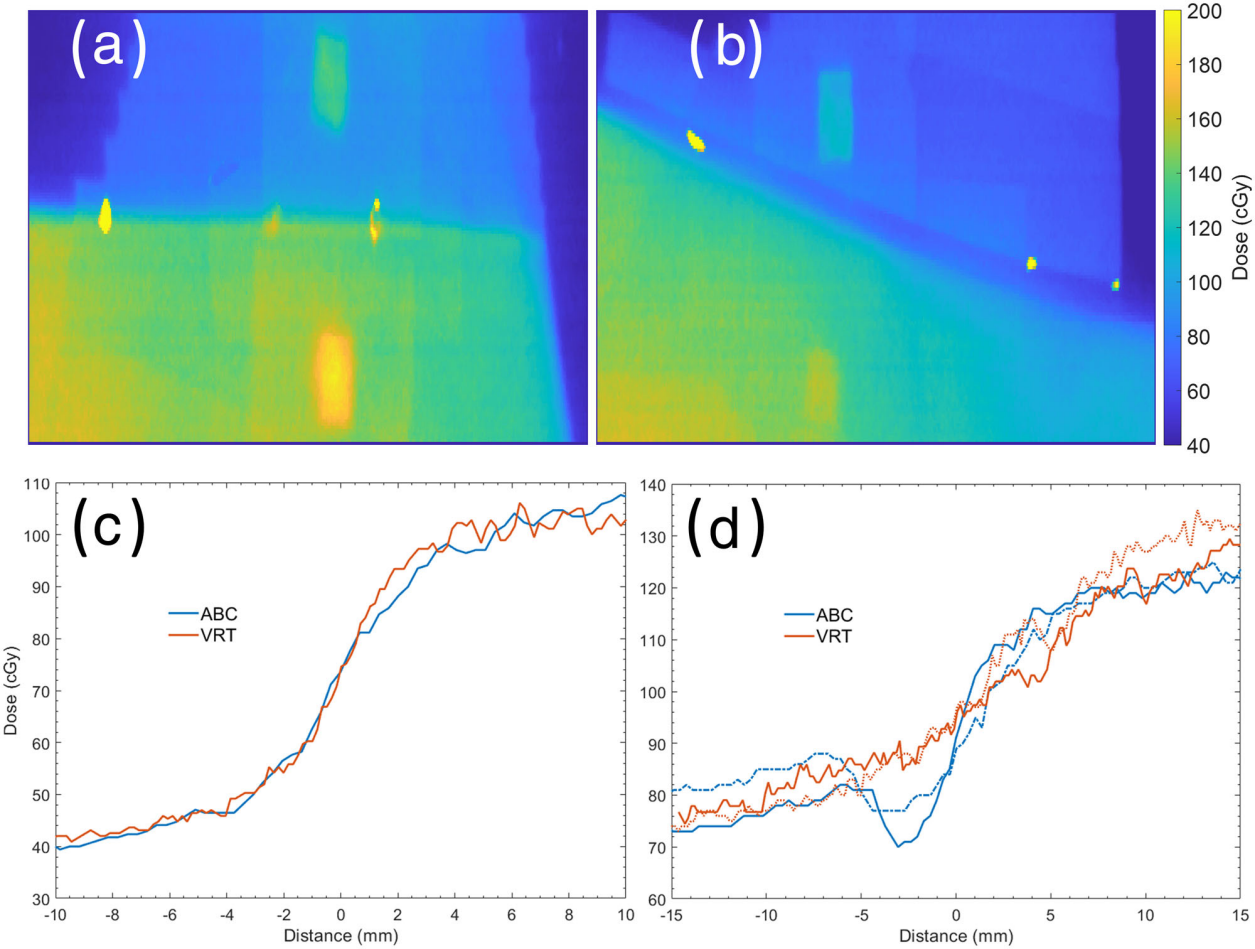
Examples of a (a) stable junction and a (b) geographic miss from film analysis at the field junction. Yellow dots mark the match line from the light field during setup. Corresponding dose profiles through the (c) stable junction and a (d) geographic miss from film dose measurements at the field junction

A total of 33 films were analyzed over five patients, 19 and 14 for ABC and VRT DIBH, respectively (5 of the 19 of the VRT needed to be excluded due to errors in film placement). None of the VRT films showed evidence of a potentially unstable junction while three fractions of the ABC films had a gap greater or equal to 3 mm between the SCV and tangents between DIBHs. Figure 5 shows an example of a stable and potentially unstable junction. Images on the left represent a stable junction, where there is a sharp dose gradient between the SCV field and the tangent fields. The images of a potentially unstable junction exhibit an underdosed gap in the junction region. These data show a similar trend to the imaging study in which both VRT and ABC can produce stable and reproducible DIBHs. However, ABC can have occasionally high intra‐fraction variability leading to a potentially unstable junction.

### 3.4 Treatment session time

Treatment session time data is reported in Table 1. Plan verify and weekly films sessions are reported separately from the daily setup and treatment delivery. Average plan verify times were 40 and 29.8 mins for ABC and VRT, respectively, among the 10 patients. Mean VRT plan verify sessions were 25% shorter in duration compared to ABC. This was not significant (n.s., *p* = 0.182) and is likely due to small sample size (*n* = 5 for each) and a larger sample is needed to accurately assess this trend. Similarly, mean weekly film sessions were 25.2 and 20.0 min for ABC and VRT, respectively (*p* = 0.041). Daily setup times were marginally shorter with mean times of 9.2 and 7.4 min for ABC and VRT, respectively (*p* = 0.007). Daily treatment duration (first field beam on to last field beam off) showed no difference with times of 9.9 and 9.7 min for ABC and VRT, respectively (n.s., *p* = 0.81). This is expected since the patient should have to take a similar amount of DIBHs to deliver the same plan. However, VRT on average, shortens the daily setup time between 15%–25% compared to ABC sessions. It is of note that the mean amount of MV imaging required for positioning throughout the entire course of treatment was almost identical for VRT and ABC at 30.5 ± 7.2 and 29.9 ± 8.7 MU, respectively. Note 1 MU was one image for setup verification.

**TABLE 1:** Time for plan verify sessions, weekly films, daily setup and daily treatment for ABC and VRT

Session	ABC (min)	VRT (min)	Difference (min)	*p*‐value
Plan verify	40.0 ± 8.0	29.8 ± 11.4	10.2	0.182
Weekly films	25.2 ± 11.0	20.0 ± 7.3	3.9	0.041
Daily setup	9.2 ± 4.4	7.4 ± 2.9	1.8	0.007
Treatment delivery	9.9 ± 3.7	9.7 ± 3.8	0.2	0.810

## 4 DISCUSSION

Multiple studies have investigated the accuracy of VRT and ABC setup individually for breast cancer patients. However, comparison between the two DIBH techniques has not been well investigated. In this study, we investigated and compared the stability and reproducibility of the two DIBH techniques for left‐sided breast cancer patients. The study consisted of two components: (1) position intrafraction stability and interfraction reproducibility study with portal images. (2) And junction dose stability with film qualitative dose measurement. The lung area data demonstrated that ABC and VRT are comparable in terms of stable and reproducibility.

The Student's *t*‐tests for the intrafraction stability concluded the difference between ABC and VRT stability was not significant (*p* = 0.76 and 0.83 for the LLat and SCV field, respectively). The mean stability across all patients was between 1.26% and 1.55%, which are consistent with other studies.^7^, ^14^ These findings demonstrated that the patient setup workflow at our institution provides good stability within a given fraction, with intrafraction stability not exceeding 3% for either modality.

The interfractional stability, while also not meeting the metric for significant did perform better using VRT compared to ABC for the LLat: 11.0 ± 3.4% and 14.9 ± 6.0% for VRT and ABC, respectively (*p* = 0.07). With patient two exceeding 28% for interfraction reproducibility for ABC compared to 15.6% for VRT. The SCV was much more reproducible compared to the LLat. This is to be expected since it should be less influenced by the BH compared the LLat, despite that patient seven had an ABC reproducibility of 34% compared to 14.3% for VRT.

**FIGURE 6:**
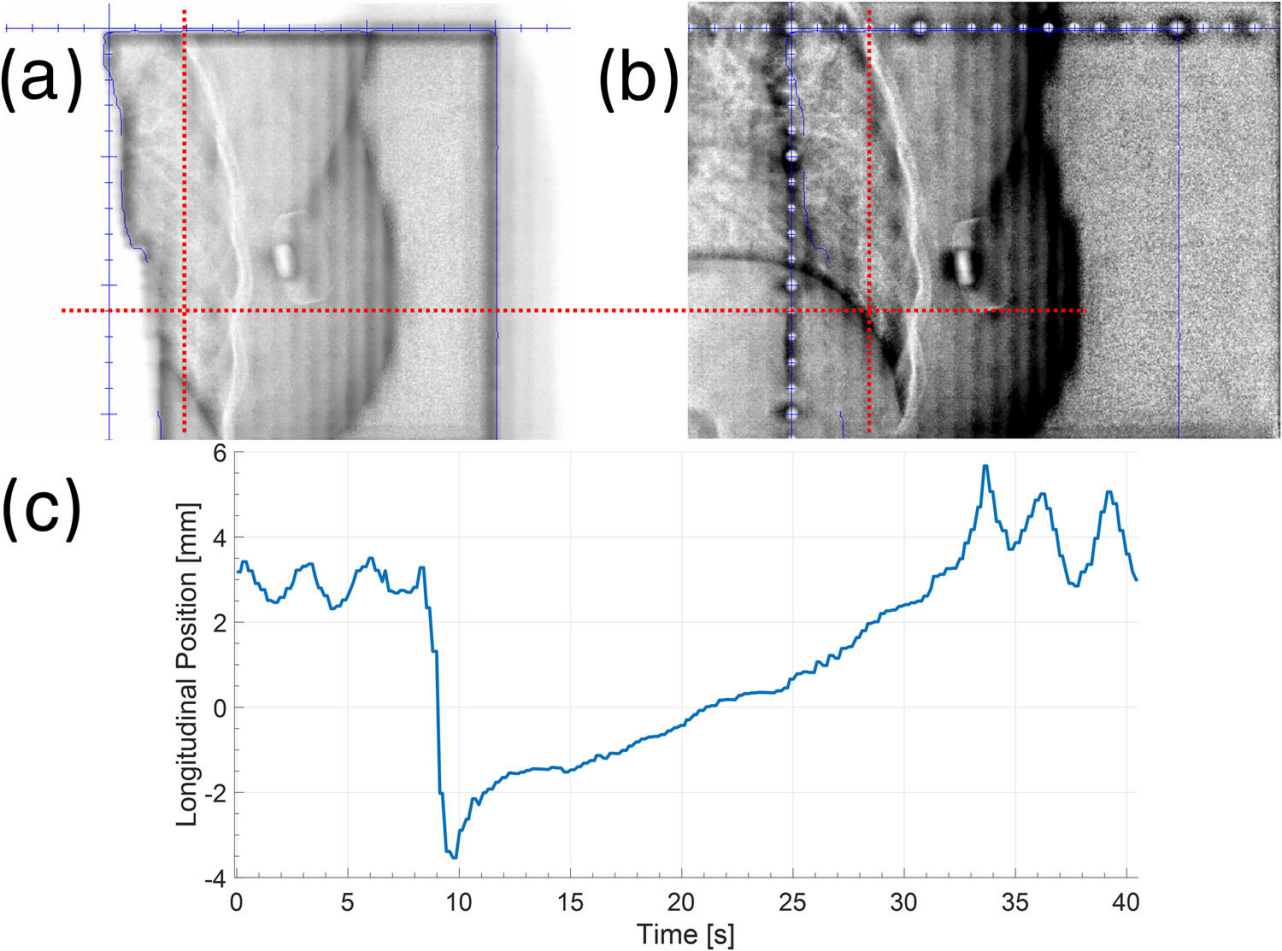
(a,b) Double exposure images showing the change in diaphragm position during a single DIBH. Dashed line is used to show the same point in each image. (c) Corresponding surface difference during this time period

A limitation of a DIBH is that the BH can drift, where patients slowly exhale during a BH. Figure 6 shows an extreme example we observed (outside the trial) in a double exposure image of right sided breast patient for DIBH with ABC. In this scenario ABC provides no indication that the patient's BH has changed, however, VRT was used with ABC to monitor the patient for future treatments as the change was readily detectable (8 mm of motion) with VRT Figure 6c. In our experience, this type of patient is rare but serves to demonstrate how VRT can aid in identifying potential mistreatments. However, this would depend on ROI choice and education of the users to identify the potential problem in switching to VRT.

The WBRT treatment planning usually allows at least 3 cm of flash to account for respiratory motion. However, depending on the direction of the motion displacements, this magnitude of stability and reproducibility can have dosimetric impact on target coverage and sparing of the organs‐at‐risk, such as the heart and the lung. Utilizing kV or 2.5 MV^15^, ^16^, ^17^, ^18^ imaging with implanted clips for alignment may offer increased accuracy at patient setup.^19^, ^20^ Further investigation of the intra‐ and inter‐fractional variations will be needed to examine the dosimetric effects.

While ABC gives the total air volume within a BH, VRT provides users with the magnitude of setup discrepancies in three directional planes. This will allow more efficient adjustments prior to the acquisition of verification EPID images. The visual feedback of the VRT system also displays the arm and chin placements, which are not possible with ABC‐based setups. Although ABC and VRT showed similar stability for treatment of left‐sided breast cancer in our study, VRT‐based alignment may be a better option in terms of patient setup efficiency and clinical workflow as shown in the time reduction in setup and plan verify (Table 1). It is also worth noting that during the trial we were relatively new to using VRT and ABC was the clinical standard for DIBH. While VRT is relatively straight forward to use, it has a learning curve for ROI design for surface tracking across a wide population and sufficient training and experience for the treating therapists to identify potential issues. Additionally, VRT requires routine education of the staff involved (physicians, physicists, and therapists). As such we expect the potential time reduction to be greater if VRT were to become the clinical standard.

## 5 CONCLUSIONS

ABC and VRT exhibited comparable stability when used for treatment of left‐sided breast cancer. The intra‐ and inter‐fractional variations of the lung position were not significantly different between fractions that were treated with ABC and VRT. For certain patients, ABC can lead to large variability due to different patient BH maneuvers, causing a dosimetric gap in the field junction. The largest benefit of VRT compared to ABC is the potential time savings with daily setup reduced between 1.8 and 3.9 min, and a potential time reduction for plan verification.

## AUTHOR CONTRIBUTIONS

David Parsons, Mindy Joo, Zohaib Iqbal, and Andrew Godley were all involved in data analysis and drafting the manuscript. Nathan Kim, Ann Spangler, Kevin Albuquerque, Amit Sawant, Bo Zhao, Xuejun Gu, and Asal Rahimi were all involved in the trial design, patient recruitment, data collection, and reviewing the draft manuscripts.

## CONFLICTS OF INTEREST

No conflicts of interest with the presented work.
